# Anterior Uveitis and Uveal Depigmentation in a Dog With Vitiligo

**DOI:** 10.1155/crve/6586766

**Published:** 2025-01-13

**Authors:** Eric C. Ledbetter, Irini D. Lamkin, Jeanine Peters-Kennedy

**Affiliations:** ^1^Department of Clinical Sciences, College of Veterinary Medicine, Cornell University, Ithaca, New York, USA; ^2^Department of Population Medicine and Diagnostic Sciences, College of Veterinary Medicine, Cornell University, Ithaca, New York, USA

**Keywords:** leukotrichia, poliosis, uveal depigmentation, uveitis, uveodermatologic syndrome, vitiligo

## Abstract

**Objective:** The objective of this study is to describe the clinical and histologic features of a dog that developed anterior uveitis and uveal depigmentation in association with vitiligo.

**Animal Studied:** A 3-year-old, female-spayed, Bernese Mountain Dog with a history of bilateral idiopathic anterior uveitis developed iris depigmentation, leukotrichia, and skin depigmentation.

**Procedures:** The initial diagnostic evaluation for uveitis was unremarkable, including general bloodwork, urinalysis, infectious disease testing, thoracic radiographs, and abdominal ultrasound. After the development of dermatologic disease, uveodermatologic syndrome was clinically suspected and cutaneous biopsy specimens were collected for histopathology.

**Results:** Cutaneous histopathology was consistent with vitiligo. Progressive and diffuse skin and hair depigmentation occurred over several years, but the dog's anterior uveitis remained well controlled on relatively minimal topical anti-inflammatory medications. No posterior segment ocular lesions developed, and the dog remained visual.

**Conclusions and Clinical Relevance:** This report indicates that anterior uveitis and uveal depigmentation can develop in dogs associated with vitiligo. The presence of bilateral uveitis and uveal depigmentation, concurrent with skin and hair depigmentation, is often considered suggestive of uveodermatologic syndrome in a dog. This report illustrates the importance of cutaneous histopathology to confirm a clinical suspicion even in the most suggestive of clinical presentations.

## 1. Introduction

Concurrent ocular and dermatologic diseases develop frequently, in part due to shared tissue embryonic origins and anatomical proximity [1]. Ocular manifestations of infectious and inflammatory dermatological diseases are particularly common in clinical practice [2]. Ocular lesions in these scenarios are varied and etiology-dependent but can include blepharitis, conjunctivitis, keratitis, and both anterior and posterior uveitis [3, 4].

Canine uveodermatologic syndrome, or Vogt–Koyanagi–Harada-like syndrome, is an infrequent condition of dogs associated with the immune-mediated destruction of melanocytes and melanin-laden tissues [5, 6]. Leukoderma, leukotrichia, erythema, and skin ulceration are observed clinically with concurrent bilateral uveal depigmentation and panuveitis [6, 7]. Vision loss from glaucoma, retinal detachment, or cataract formation is common with canine uveodermatologic syndrome, and aggressive topical and systemic immunosuppressive therapy is generally indicated [8]. Histopathologic evaluation of cutaneous biopsy specimens or enucleated globes is required to definitely confirm a diagnosis of uveodermatologic syndrome [9]. Despite this, it is frequently stated that bilateral uveitis combined with dermatologic abnormalities and uveal depigmentation should be considered highly suggestive of uveodermatologic syndrome. As a result, some dogs with characteristic clinical lesions are likely treated presumptively for uveodermatologic syndrome in the absence of confirmatory histopathology results [8, 10].

Vitiligo is an uncommon, acquired canine disease associated with the destruction of melanocytes resulting in leukoderma and leukoctrichia [11]. Clinical lesions in dogs with vitiligo are typically noninflammatory, and ocular disease is not considered a typical finding [12]. Vitiligo is frequently believed to be a purely cosmetic issue in dogs, and treatment is often not recommended. The present report describes a case of histologically confirmed canine vitiligo associated with anterior uveitis and uveal depigmentation that clinically masqueraded as uveodermatologic syndrome. The described case illustrates the importance of pursuing a histopathologic diagnosis in cases of suspected canine uveodermatologic syndrome and the need to evaluate dogs with vitiligo for ocular disease.

## 2. Case Description

Informed owner consent to use all collected clinical information for research and publication purposes was obtained in writing for the dog described in this report. A 3-year-old, female-spayed Bernese Mountain Dog was referred to the Cornell University Hospital for Animals Ophthalmology Service for evaluation of red eyes first noted several weeks prior by the client. The referring veterinarian started treatment with neomycin–polymyxin b–dexamethasone solution (q12h) in both eyes (OU) 4 weeks prior, but the client administered the medication inconsistently and observed minimal improvement. The dog had no significant previous medical history and was otherwise apparently healthy.

Complete ophthalmic examination, including slit-lamp biomicroscopy (Kowa Co, Tokyo, Japan), indirect ophthalmoscopy (Heine USA Ltd, Dover, New Hampshire) before and after induction of pharmacologic mydriasis (tropicamide 1% solution, Bausch & Lomb, Tampa, Florida), Schirmer I tear tests, intraocular pressure evaluation by applanation tonometry (Reichert Inc, Depew, New York) after application of topical anesthetic (proparacaine hydrochloride 0.5% ophthalmic solution, Bausch & Lomb), and ocular surface fluorescein staining (Akorn Inc, Lake Forest, Illinois), were performed. Palpebral reflex, pupillary light reflex, and menace responses were present in OU. Schirmer I tear tests were 25 in the right eye (OD) and 21 in the left eye (OS) (millimeters per minute, approximate canine reference range: 15–25 mm/min). Intraocular pressures were 13 in the OD and 16 in the OS (millimeters of mercury). Intermittent blepharospasm and mild conjunctival hyperemia were present in OU. Multifocal, faint subepithelial white crystalline deposits were present in the axial cornea in OU. There were trace aqueous flare and cell present in the anterior chamber in OU. Multifocal posterior synechiae and dyscoria were observed in OU with mild peripheral iris thickening in the OD. Multifocal, punctate pigment was present on the anterior lens capsule in OU. The fundus was unremarkable in OU.

No additional abnormalities were identified on complete physical examination. Results of complete blood count, serum biochemistry panel, and urinalysis were unremarkable. Tick-borne disease testing (SNAP 4Dx Plus, IDEXX Laboratories Inc., Westbrook, Maine) and *Brucella canis* serology (New York State Animal Health Diagnostic Center, Ithaca, New York) were negative. Thoracic radiographs and abdominal ultrasound detected no clinically significant abnormalities. The dog was diagnosed with idiopathic, chronic anterior uveitis and discharged receiving prednisolone acetate 1% solution (q12h OU) and diclofenac 0.1% solution (q12h OU). Medications were to be administered until the recommended recheck evaluation in 3–4 weeks. The client noted rapid improvement in blepharospasm and hyperemia, so they discontinued the ophthalmic medications after 4 weeks and did not return the dog for evaluation until 8 weeks later when clinical signs returned.

**Figure 1:**
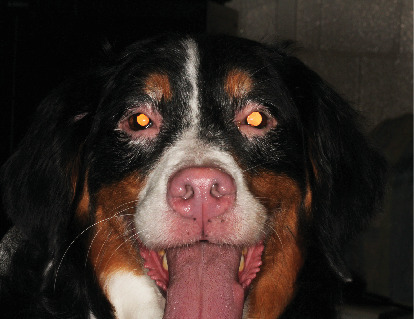
Clinical photograph of a Bernese Mountain Dog with vitiligo, uveal depigmentation, and anterior uveitis. Periocular alopecia, skin depigmentation, and leukotrichia are present in OU. Skin depigmentation of the lips, nasal bridge, and nasal planum is present with multifocal areas of bilateral pinnal leukotrichia.

**Figure 2:**
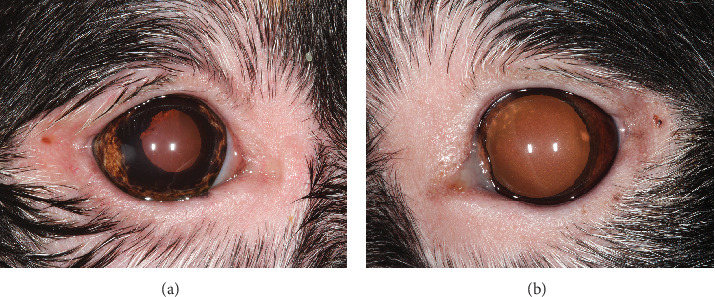
Clinical photograph of the right (a) and left (b) eyes of a Bernese Mountain Dog with vitiligo, uveal depigmentation, and anterior uveitis. Periocular alopecia, skin depigmentation, and leukotrichia are present in OU. Multifocal, faint subepithelial white crystalline deposits are present in the axial cornea in OU. Multifocal posterior synechiae and dyscoria are observed with peripheral iris depigmentation in OU. Multifocal, punctate pigment is present on the anterior lens capsule in OU.

Upon recheck examination 8 weeks after the initial presentation, focal periocular skin and hair depigmentation were present in OU. In addition, skin and hair depigmentation was noted around the lips, nose, and muzzle (Figure 1). Palpebral reflex, pupillary light reflex, and menace responses were present in OU. Schirmer I tear tests were 20 in the OD and 17 in the OS (millimeters per minute). Intraocular pressures were 12 in the OD and 17 in the OS (millimeters of mercury). Intermittent blepharospasm and moderate conjunctival hyperemia were present in OU. The crystalline corneal deposits were unchanged in OU. Mild aqueous flare and cell were present in OU. Multifocal posterior synechiae and dyscoria persisted in OU but were now associated with peripheral iris depigmentation confined to the ciliary zone of the iris (Figure 2). Multifocal punctate pigment was present on the anterior lens capsule, and the fundus was unremarkable in OU. Based upon signalment and clinical findings, a diagnosis of uveodermatologic syndrome was suspected. Cutaneous biopsies and evaluation by the Cornell University Dermatology Service were recommended for confirmation and scheduled. The dog was discharged receiving prednisolone acetate 1% solution (q12h OU), diclofenac 0.1% solution (q12h OU), and prednisolone (1.5 mg/kg PO q24h) until the recheck examination.

Ophthalmic examination was repeated 1 week later (9 weeks from the initial presentation) immediately prior to the dermatologist evaluation. No hyperemia or aqueous flare was present in OU, but the ophthalmic examination was otherwise substantially unchanged including a normal fundus evaluation in OU. Dermatologic examination identified periocular alopecia and depigmentation with focal areas of leukotrichia dorsomedial to OU. Depigmentation of the lips, nasal bridge, and perianal region was noted. Complete nasal planum depigmentation with mild loss of the cobblestone architecture dorsally and mild, multifocal areas of leukotrichia were present at the pinnal margins bilaterally. No other dermatologic abnormalities were identified, and cutaneous biopsy specimens were collected under general anesthesia from the superior eyelid in the OD, the right corner of the nasal planum, and the lower right lip. Pending histopathology results, the dog was discharged receiving the same previous medications with the addition of mycophenolate (15 mg/kg PO q12h) for additional control of presumptive uveodermatologic syndrome.

**Figure 3:**
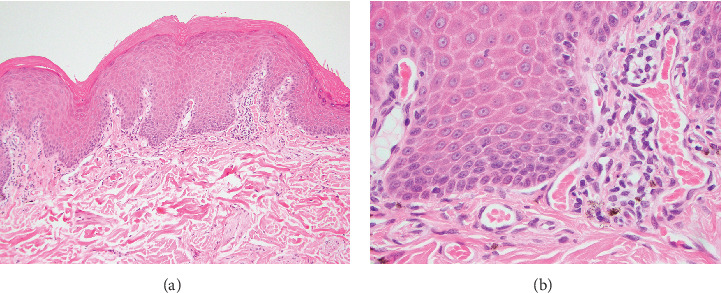
Low- (a) and high-magnification (b) histopathologic photomicrographs of nasal planum cutaneous biopsies in a dog with vitiligo. There is a marked reduction in epidermal melanin. A small number of lymphocytes and melanophages surround capillaries in the superficial dermis. A small number of lymphocytes migrate into the overlying epidermis. Stained with hematoxylin and eosin. Original magnification 100× (a) and 400× (b).

Histopathology results were received 1 week later. Mild, multifocal, chronic, lymphocytic, histiocytic, superficial, and perivascular dermatitis with mild pigmentary incontinence and segmental epidermal depigmentation were present in all evaluated samples (Figure 3). The histologic findings of very mild inflammation accompanied by degrees of segmental to diffuse depigmentation and pigmentary incontinence in all examined sections were consistent with vitiligo. The mycophenolate was discontinued, and the dog was slowly tapered off the oral prednisolone over a 2-week period. Topical ophthalmic medications were continued. The cutaneous biopsy sites healed without complication. The dog developed partial anorexia and diarrhea after the biopsy procedure, which resolved rapidly once the mycophenolate was discontinued.

The dog was reexamined 3 weeks later (12 weeks from the initial presentation). No hyperemia or aqueous flare was present in OU. The ophthalmic examination was otherwise substantially unchanged in OU, including the appearance of the peripheral iris depigmentation and periocular skin depigmentation and leukotrichia. The dog was discharged receiving prednisolone acetate 1% solution (q24h OU for 6 weeks then discontinued) and diclofenac 0.1% solution (q12h OU). A recheck evaluation was recommended in 8 weeks, but the client did not return for 6 months.

The dog was presented for a 2-day history of acute-onset blepharospasm 6 months later (9 months from initial presentation). The client communicated that, apart from progressive skin and hair depigmentation, the dog had been doing well and they discontinued the diclofenac solution several weeks earlier. The referring veterinarian restarted treatment with prednisolone 1% solution (q12h OU) and diclofenac 0.1% solution (q12h OU) in response to the relapse of clinical signs prior to referral, and the dog's clinical signs rapidly improved. During ophthalmic examination, no blepharospasm or conjunctival hyperemia was present in OU. Aqueous flare was absent, but 1+ aqueous cell was present in OU. Intraocular pressures were 12 in the OD and 13 in the OS (millimeters of mercury). The crystalline corneal deposits had mildly progressed in OU. The multifocal posterior synechiae, dyscoria, and peripheral iris depigmentation were static in OU. The remainder of the ophthalmic exam was unchanged in OU, including a normal fundus examination.

Periodic examinations and communications with the client were performed over the subsequent 2.5 years by both the authors and referring veterinarians. Progressive and near complete skin and hair depigmentation occurred during this period. No ophthalmic clinical signs were noted by the client or referring veterinarian when both prednisolone and diclofenac were administered (q12h); however, repeated attempts by the referring veterinarian to reduce the frequency of the ophthalmic medication resulted in episodes of blepharospasm that quickly resolved with reintroduction of the medications.

## 3. Discussion

Bernese Mountain Dogs are reported to develop both vitiligo and uveodermatologic syndrome [11, 12]. The presence of bilateral uveitis and uveal depigmentation, concurrent with skin and hair depigmentation, might be considered strongly suggestive of uveodermatologic syndrome in a dog from a breed with known susceptibility to the condition. This report emphasizes the importance of cutaneous histopathology to confirm a clinical suspicion even in the most suggestive of clinical presentations. Characteristic skin histopathologic findings in dogs with uveodermatologic syndrome are a lichenoid pattern of inflammation with abundant melanin-laden macrophages [13]. Conversely, in dogs with vitiligo, skin histopathology displays mild or absent inflammation accompanied by segmental to diffuse depigmentation, pigmentary incontinence, and epidermal architecture is retained [14]. As occurred in the described dog, achieving a correct and definitive diagnosis provides clients with a more accurate long-term visual prognosis while avoiding unnecessary treatments and the potential adverse effects associated with systemic immunosuppressive therapeutics. A definitive diagnosis will also permit one to avoid, in individual dogs and clinical studies, erroneously attributing treatment successes for canine uveodermatologic syndrome to a particular medication or therapeutic regimen.

Notable clinical differences between the described dog with vitiligo and typical canine uveodermatologic syndrome cases include the lack of posterior segment ocular involvement and the relatively minimal anti-inflammatory treatment required to control the dog's uveitis long term [8]. The dog was successfully treated with a combination of a topical corticosteroid and nonsteroidal medication. Initially, this combination was selected with the plan of tapering off the prednisolone and using diclofenac for long-term maintenance as soon as possible to avoid complications associated with long-term corticosteroid use (including potential exacerbation of the crystalline corneal opacities) [15]. The dog's uveitis and clinical signs remained well controlled on this minimal topical medication protocol, and recurrences of ocular disease only occurred when medications were discontinued or tapered. In addition, mucocutaneous erosions are occasionally reported in dogs with uveodermatologic syndrome and were absent in the described case.

The pathogenesis of canine and human vitiligo is poorly understood, but it is hypothesized to be a multifactorial disease involving both autoimmune mechanisms and additional factors (e.g., genetic susceptibility, oxidative damage, trauma, and stress) [16]. An initiating event is speculated to damage melanocytes, induce novel autoantigens, expose cryptic cellular antigens, and activate immune responses against the melanocytes [17]. Canine vitiligo typically begins as multifocal, depigmented macules or patches on the face [18–20]. The gingiva and lips are the most common initial sites for lesion development. As the disease progresses, depigmentation generally remains confined to the head with the eyelids, eyelashes, nasal planum, oral cavity, pinnae, and muzzle the most frequently involved regions [18–20]. Less commonly, depigmentation may spread to other anatomic regions (e.g., footpads, scrotum, nails, paws, limbs, neck, trunk, and rump) or become generalized [21, 22].

The occurrence of iris depigmentation associated with generalized skin and hair depigmentation in a single Australian shepherd with vitiligo is briefly described in a figure legend of a review article [11], but to the authors' knowledge, the presence of associated anterior uveitis has not been previously reported in dogs with histopathologically confirmed vitiligo. In humans with vitiligo, eyelid depigmentation, eyebrow poliosis, eyelash poliosis, iris hypopigmentation, iris atrophy, hypopigmented trabecular meshwork, retinal pigmentation epithelial depigmentation, and choroidal depigmentation are relatively common ocular lesions [23–25]. Iridocyclitis, chorioretinitis, and keratoconjunctivitis sicca are also associated with vitiligo in human patients but are considered relatively infrequent, and the pathophysiological relationship between these conditions remains unclear [24–27].

Additional research into the frequency and characteristics of ocular lesions in dogs with histopathologically confirmed vitiligo is warranted. In addition, investigations into the potential genetic contributions of this conditions in dogs, as have been described for other oculocutaneous syndromes, should be pursued [28].

## Data Availability

The data that support the findings of this study are available from the corresponding author upon reasonable request.
